# The Sound of Silence: Minorities, Abstention and Democracy

**DOI:** 10.3390/e24010056

**Published:** 2021-12-28

**Authors:** Alessio Emanuele Biondo, Alessandro Pluchino, Roberto Zanola

**Affiliations:** 1 Department of Economics and Business, University of Catania, 95129 Catania, Italy; ae.biondo@unict.it; 2 Department of Physics and Astronomy, University of Catania and INFN-Section of Catania, 95123 Catania, Italy; 3 Department of Law and Political, Economic, and Social Sciences, University of Eastern Piedmont, 15121 Alessandria, Italy; roberto.zanola@uniupo.it

**Keywords:** democracy, opinion dynamics, turnout, majority, regime

## Abstract

Despite the existence of an extensive literature, no definitive conclusion seems to emerge on the extent to which minorities are guaranteed by democratic rules in political systems. This paper contributes to this debate by proposing a modified Heigselmann and Krauss two-dimensional model of preferences in order to capture the role of abstention on minority representativeness. Regardless of the typology of abstention, simulation results show that voter abstention always benefits minorities.

## 1. Introduction

In democratic systems the formation of consensus is a challenging issue for political science. In particular, opinion polarization is considered a crucial factor in the consensus formation process [1,2,3]: on one hand, if present to a moderate extent, it seems to favor some democratic features such as, for example, turnout [4]; by contrast, higher levels of polarization can amplify opinion fragmentation, preventing the achievement of any compromise among parties [5].

Actually, democracy is a complex, self-organizing adaptive system, which involves many actors (voters, parties, governments) interacting with each other in a nonlinear way. Thus, the exploration of the decision making process is a hard task and, over the years, has attracted the attention of scientists from different disciplines, including physical sciences. In the last decades, complexity science and physics-inspired scholars have developed new theoretical and computational models to analyze socioeconomic systems, providing policy makers with new tools to manage with disasters, crime, terrorism, migrations, urban development and traffic, war, financial markets, disease spreading, etc. (for extended reviews see [6,7,8]).

In this context, simple mathematical models of opinion dynamics allow to investigate the interplay between polarization and consensus in sociocultural systems. Among them, *bounded-confidence* models are able to well reproduce the main stylized facts of the process of consensus formation by tuning the size of a tolerance interval for the opinion interactions [9,10,11]. In particular, the Hegselmann and Krause model [10] stimulated many other scholars to develop the original version to take account of different aspects of the convergence towards consensus in a given population [12,13,14].

In the political framework, opinion dynamic models also appear to be the best candidates to simulate voting dynamics and competitions among parties in democratic systems [15,16,17]. Democratic institutions assume significant decisions by voting on them. Although the existence of an undefined number of different voting systems, two main families dominate the relevant literature for the case in which people are asked to vote for choosing among several alternatives: the plurality rule, which selects the alternative most preferred by the greatest number of voters, and the majority rule, where the winner receives more than half of the votes. In both cases, a concern for the representativeness of minority emerges.

A plurality rule is the simplest way of selecting an alternative, but at the cost of non-plurality voters, who could feel displeased by the final decision. In [18], it is suggested that new alternatives (especially centralized located in the preference setting) have difficulties in winning and tend not to be considered as serious challengers or viable alternatives, forcing people to vote for extremist alternatives or to abstain. When the alternatives are three, plurality rule forces voters, who prefer the alternative expected to come in third, to have incentive to abandon it and vote for their second favourite alternative, the so-called Duvergers law [19]. This implies both that there can always be polarisation (anything goes) and that there will always be misaligned voting since people will vote for an option which is not their most preferred [20]. These limits are discussed in [21], where a panel of voting theorists deemed plurality as the worst voting rule.

To overcome these criticisms, alternative mechanisms, such as voting by an absolute majority is used. It is commonly argued that the majority rule favors political equality and an efficient coordination of voter preferences to outcomes [22]. Additionally, it has been claimed [23] that majority rule allows minorities to form a coalition that can “overturn an unacceptable outcome”, without privileging some minority over every others. Analogously, “filtering mechanism of deliberation, representation, and coalition-building”, which characterizes American states with tradition democratic institutions, has been shown to protect the rights of minorities [24]. More recently, it has been suggested that democracy is an instrument to contrast populism since it allows to protect minorities against injustice [25]. However, there is also a potential drawback of the majority rule, the risk for the minority not to be adequately protected in the absence of a system with external checks and balances, i.e., the so-called majority tyranny [26].

Hence, irrespectively of the voting system, both plurality and majority rules could negatively impact on minority representativeness. However, minority is not the only dimension to take account of when discussing of the representativeness of different democratic voting mechanisms. During several years scholars have almost omitted to analyze the impact of voters’ abstention on the selected alternative [27]. An exception is represented, for example, by Dougherty and Edward [28], who compared majority vs. plurality rules using different criteria to analyze which rule fits them better. Depending on the results, votes to abstain could be considered as votes against the winning alternative or out of the tally. Thus, in democratic systems the option to abstain is one that can undoubted impact on the way the final decision benefits minorities.

This paper aims to explore this issue by simulating the role of voters’ abstention on minorities representativeness in a one-shot voting process. Specifically, we propose a modified two-dimensional Hegselmann and Krause bounded confidence model [10] in order to explore the role of voters’ absention on minorities representativeness in a one-shot voting process. Two different types of voter abstention are analyzed: strategic and non-strategic. The first type refers to people who rationally delegate the decision to more informed voters, even if voting is costless (a phenomenon called *swing voter’s curse* [29]); the second one, also described as protest abstention [18], refer to people who are uncertain on alternatives and consequently abstain since the ’expected benefits of voting are low’.

The current study makes a number of contributions to the existing literature. Firstly, the simulation of the dynamics of preferences allows to capture the voters’ level of satisfaction/disappointment about the final decision, which is a crucial dimension to evaluate whether a majority tyranny does exist. Secondly, we explicitly investigate the role of absenteeism on voting process, which significantly enriches the analysis of effects of majority decisions on minorities. Specifically, regardless of the typology of abstention, simulation results show that voter abstention always benefits minorities.

The remainder of the paper is organized as follows: in Section 2 the model is described; in Section 3 results of simulations are discussed; finally, main conclusions are summarized in Section 4.

## 2. The Model

We model a set of *N* voters as points distributed within a two-dimensional preference space $\Pi =\left[0,1\right]\times \left[0,1\right]$, where each voter is defined by her preference profile vector $x_{i}=\left[x_{i,1},x_{i,2}\right],i=1,2,\cdots ,N$, with $x_{i,1}$ and $x_{i,2}$ continuous coordinates. Such a setup is characteristic of the 2D version of the Hegselmann and Krause (HK) model, that we decided to adopt as starting point for our model since its behavior is richer than that of the original 1D counterpart [30]. In the political context we are considering here, the two coordinates could represent, for example, the voter’s opinions about, respectively, targets and tools of a certain policy on which the assembly must make a decision.

**Figure 1:**
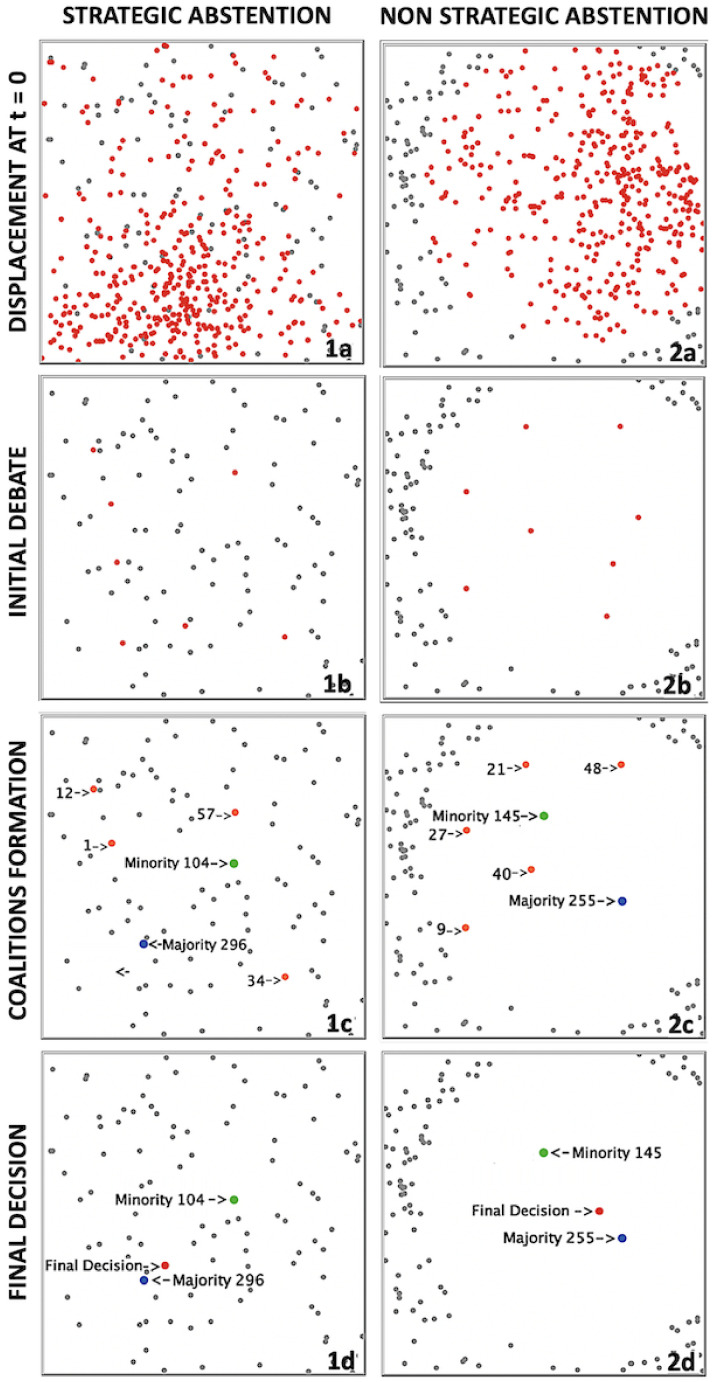
Subsequent phases of a single simulation run (N=500, $p_{A}=20\%$, f=0.5). Column 1: strategic abstention; Column 2: non-strategic abstention. Panels (**a**): random initial conditions with preferential displacement (abstained voters in gray, active voters in red). Panels (**b**): in red are visible the stationary clusters formed by active voters at the end of the debate phase. Panels (**c**): some clusters have collapsed in the majority coalit ion (blue point) while the others (orange points), considered all together, represent the minority coalition (green point). Labels indicate the size of both clusters and coalitions. Panels (**d**): all the existing clusters have collapsed in only one (red point), representing the position of the final decision. See text for more details.

The simulation process consists of an opportune modification of the original Hegselmann and Krause dynamics, specifically designed for virtually reproducing the democratic procedure which drives an assembly of voters towards a final political decision. This process will be implemented through the following subsequent phases:

a Initial Conditions (t=0). At the beginning of each simulation run the *N* voters are distributed in the preference space, adopting a procedure inspired by the *preferential attachment* algorithm of Barabasi and Albert [31], that we call “preferential displacement”. Specifically, we insert each new voter in a random position of the entire space with a probability of 30%, while the same voter is randomly displaced within the δ-neighborhood of an existing voter *i* (i.e., within a circular domain centered in $\left(x_{i,1},x_{i,2}\right)$ with radius equal to δ) with a probability of 70%. The latter voter is selected with a probability $\pi_{i}\left(\delta \right)$ proportional to the fraction of other voters already included in the same neighbourhood. In other words, $\pi_{i}\left(\delta \right)=n_{i}/\sum_{j}n_{j}$, being $n_{i}$ the number of voters present in the δ-neighborhood of *i* and $\sum_{j}n_{j}$ the total number of voters already displaced in the preference space. At variance with the standard uniform initial conditions, typical of the HK model, this process will produce an asymmetric distribution of voters, which will tend to visibly aggregate inside a certain (randomly selected, different for each simulation run) region in the preference space. The preferential displacement, on one hand, makes the initial conditions intuitively more realistic, since it is likely that voters’ opinions about the policy under discussion could concentrate from the beginning around a specific combination of target and tools; on the other hand, it will also result to be essential for the Majority formation, as explained later (see step *c*).
  Another important feature of the model is that not all the agents are necessarily involved in the voting process: as a matter of fact, a certain percentage $p_{A}$ of them can decide to abstain from the beginning. These $N_{A}$ abstained voters neither move nor interact with the other active voters, thus remaining in their initial positions in the preference space for the whole simulation. Since, as discussed before, we would like to model two types of voter abstention, the strategic one (swing voter’s curse) and the non-strategic one (protest abstention), two different ways of selecting the abstained voters in the preference space will be implemented: strategic abstainers will be randomly selected among the *N* voters, while non-strategic ones will be chosen at margins of the preference space, surrounding active voters. As we will show, though non interacting with the active voters, abstainers will still influence the dynamics of the debate phase just thanks to their presence and to their different placement in the preference space.
  In panels 1a and 2a of Figure 1 we show an example of initial conditions for two different simulation runs, with N=500 voters each and with a percentage $p_{A}=20\%$ of abstention. Voters are arranged according with the preferential displacement procedure: together with the 400 active voters (red points), one can find the $N_{A}=100$ abstainers (gray points) who either are randomly distributed in the preference space, if their abstention is strategic (1a), or surround the active voters, if their abstention is non-strategic (2a).
b Debate Process ($t\in (0,t_{D}]$). In the debate phase, starting at t>0, we implement a standard (discrete) HK dynamics in two-dimensions involving only the $N-N_{A}$ active voters. Each active voter *i*-th is endowed with a *compatibility domain*, $B(x_{i},\varepsilon )$, defined as a circle centered on her preference profile $x_{i}$, with radius equal to the so-called *confidence bound*, ε∈[0,1], which in this phase is the same for all the voters. The political debate is modeled as the parallel update, at each time step t+1, of all the voters’ profiles, so that each of them becomes equal to the average of the preference profiles of all the active voters included within her compatibility domain at time *t*. In other words:
  (1) $$x_{i}\left(t+1\right)=\frac{\sum_{j:∥x_{i}\left(t\right)-x_{j}\left(t\right)∥<\varepsilon }a_{ij}x_{j}\left(t\right)}{\sum_{j:∥x_{i}\left(t\right)-x_{j}\left(t\right)∥<\varepsilon }a_{ij}}\;\;\;\;\left(i=1,\cdots ,N\right)$$
  where $∥x_{i}\left(t\right)-x_{j}\left(t\right)∥$ is the metric distance between the preference vectors *i*-th and *j*-th, and $a_{ij}$ is the adjacency matrix of the graph which models the community of the *N* interacting agents in the real world. In this study, we consider a complete graph, i.e., a community of individuals connected all to each other, therefore $a_{ij}=1$ for i≠j and $a_{ij}=0$ for i=j. The decisional process is represented by the route of convergence towards one or more non-reducible *steady states*, which produces different outcomes according to the value of the confidence bound. Below a critical threshold $\varepsilon_{c}$, it asymptotically generates a non-reducible state with $n_{\chi }\in \left[2,N\right]$ clusters of preferences, such that $\lim_{\varepsilon \to 0}n_{\chi }=N$ and $\lim_{\varepsilon \to \varepsilon_{c}}n_{\chi }=2$. Above such a critical threshold, consensus is always achieved, i.e., $n_{\chi }=1$, independently of the initial preferences distribution. In [32], it has been shown that the value of $\varepsilon_{c}$ in the 1DHK model strictly depends on the type of considered graphs: for a complete graph, one has $\varepsilon_{c}=0.2$. In [30], it has been found a similar threshold also for the 2D version of the HK model, thus in the following we definitely assume $\varepsilon_{c}=0.2$.
  In order to calibrate the confidence bound, we introduce a parameter f∈(0,1), called “political fragmentation”. By defining $\varepsilon \left(f\right)=\frac{\varepsilon_{c}}{2}\left(2-f\right)$, the confidence bound can vary below its critical threshold in the interval $\left[\frac{\varepsilon_{c}}{2},\varepsilon_{c}\right]$, thus producing few clusters when f→0 and many clusters when f→1. In presence of non-strategic abstention, the confidence bound is further reduced according with the following expression: $\varepsilon \left(f,p_{A}\right)=\varepsilon \left(f\right)\frac{100-p_{A}}{100}$, in order to take into account the reduction of the preference space occupied by active voters. The size and the symmetry of the clusters of voters obtained at the end of the debate phase depend on both their initial distribution over the preference space and the degree of abstention. In panels 1b and 2b of Figure 1 we show the aspect of the preference space at time $t_{D}$ for the two considered simulation runs, where we set f=0.5: the system has reached its steady state with all the active voters collapsed in several clusters with different sizes for both types of abstention.
c Majority Formation ($t\in (t_{D},t_{M}]$). After the debate phase, we need some of the newly formed clusters to merge in order to realize some majority. Such a goal can be realized by rescaling, at $t=t_{D}$, the confidence bound of voters in each cluster as $\varepsilon_{i}=\varepsilon \left(f,p_{A}\right)+\left[0.5-\varepsilon \left(f,p_{A}\right)\right]\frac{N_{C}}{N}$, being $N_{C}$ the size of the cluster to which voter *i* belongs. In other words, voters belonging to larger clusters will interact with a greater number of other voters, and viceversa. The process goes on until a new steady state is reached at $t=t_{M}$, where some clusters have collapsed to form the relative majority *M*, whose position $x_{M}$ is represented by blue points in panels 1c and 2c of Figure 1; the remaining clusters (orange points) are considered together to form the relative minority *m* (green point), whose position $x_{m}$ in the preference space coincides with the weighted average of the positions of all the clusters contributing to it. Of course we must require that the size $N_{M}$ of majority is always greater than the size $N_{m}$ of minority, i.e., $N_{M}>N_{m}$: as anticipated, we verified that this can be realized only with “preferential displacement” initial conditions, which moves the center of gravity of preferences away from the geometric center of the space, thus helping the majority formation (anyway, in case this condition should be still not fulfilled, we stop the simulation and repeat the run starting from new initial conditions).
d Final Decision ($t\in (t_{M},t_{F}]$). At the beginning of this last phase the confidence bound of all the voters is set at $\varepsilon_{i}=0.5$, well above the critical threshold. Therefore, the system quickly moves towards a steady state where all the active voters have collapsed into a single cluster, which represents the final decision emerging (through the voting process) from the compromise between their respective preferences within the two coalitions. The position $x_{F}$ of such a cluster, reached at time $t=t_{F}$, typically lies somewhere between the positions of the majority and the minority (red point in panels 1d and 2d of Figure 1), depending on both the relative size of the two coalitions and their relative positions at $t=t_{M}$ within the preference space.

At the end of the simulation run, i.e., once reached the final decision at $t=t_{F}$, we need to quantify the distortion of preferences for both majority and minority, in order to compare them and evaluate minority representativeness. To achieve this goal, we introduce what we call the “Global Preference Shift” (gps), defined as follows:

- For the majority component (M) it is the metric distance between the final position (i.e., the representative preference profile) of the majority cluster and that of the final decision: ${\mathrm{GPS}}_{M}=∥x_{M}-x_{F}∥$;
- For the minority component (m) it is the metric distance between the final position (i.e., the representative preference profile) of the minority cluster and that of the final decision: ${\mathrm{GPS}}_{m}=∥x_{m}-x_{F}∥$;

In general, we would expect that ${\mathrm{GPS}}_{M}$ will be smaller than ${\mathrm{GPS}}_{m}$, since the weight of the majority coalition in “attracting” the position of the final decision in the preference space is bigger by definition due to its greater size ($N_{M}>N_{m}$). However, the exact extent of the difference between these preference shifts does emerge in a complex and unpredictable way from the whole dynamical process, making the results slightly different for different simulation runs and strictly depending not only on the random realization of the initial conditions, but also on the values of the main control parameters (fragmentation level, type and degree of abstention). Therefore, the question about the possible existence of a tyranny of majority for a political system, with a given fragmentation level and in presence of a certain degree of strategic or non strategic abstention, remains open and needs a more extensive simulation campaign to be answered to.

## 3. Simulation Results

In this section, we present the simulation results for a community of N=500 voters. For different combinations of fragmentation level and degree of abstention, we analyze the relative size of majority (M) and minority (m), distinguishing between simple majority (the majority of the $N-N_{A}$ active voters) and absolute majority (N/2+1 of voters). We perform over 1000 Monte Carlo repetitions (simulation runs) for each combination of the control parameters to have statistically significant results, independent from the initial displacement of voters in the preference space. Specifically, three different values of political fragmentation are analyzed, namely f=0.1, f=0.5 and f=0.9, and for each of them we consider four increasing degrees of abstention ($p_{A}=0\%,10\%,30\%,50\%$).

**Figure 2:**
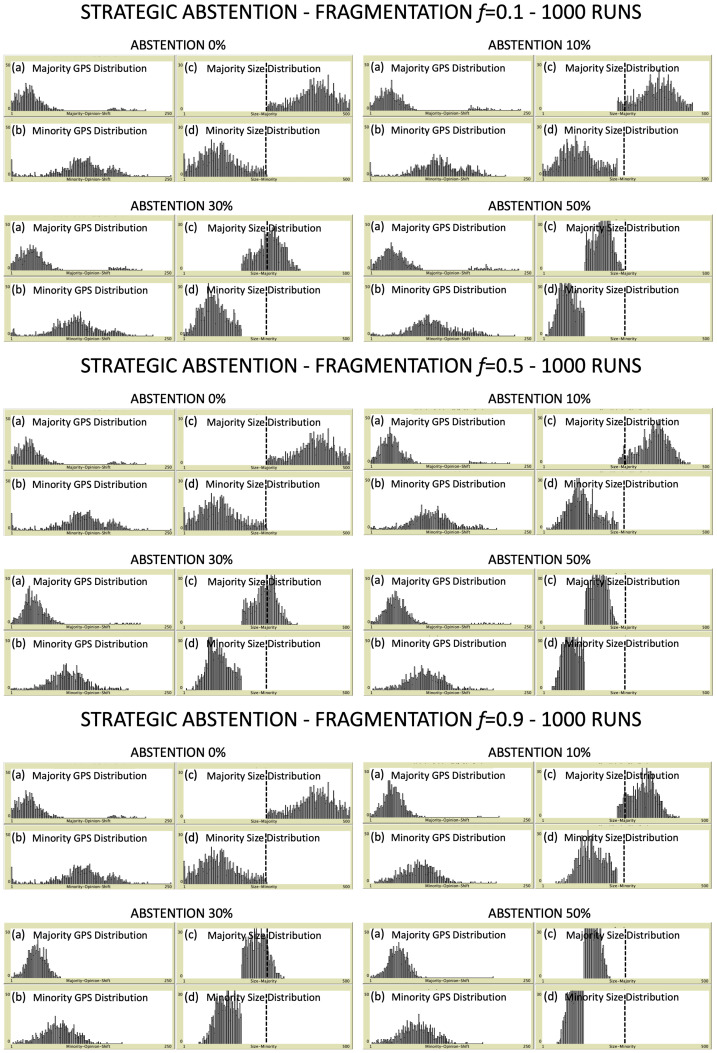
Strategic abstention—In correspondence of each fragmentation level and each degree of abstention, the following four panels are reported: (**a**) majority gps distribution; (**b**) minority gps distribution; (**c**) majority size distribution; (**d**) minority size distribution. Output data are collected over 1000 simulation runs. Scale of *y*-axis goes from 0 to 50 for GPS distributions and from 0 to 30 for size ones. The absolute majority threshold at N/2=250 voters is reported in panels (**c**,**d**) as a vertical dashed line. See text for more details.

**Figure 3:**
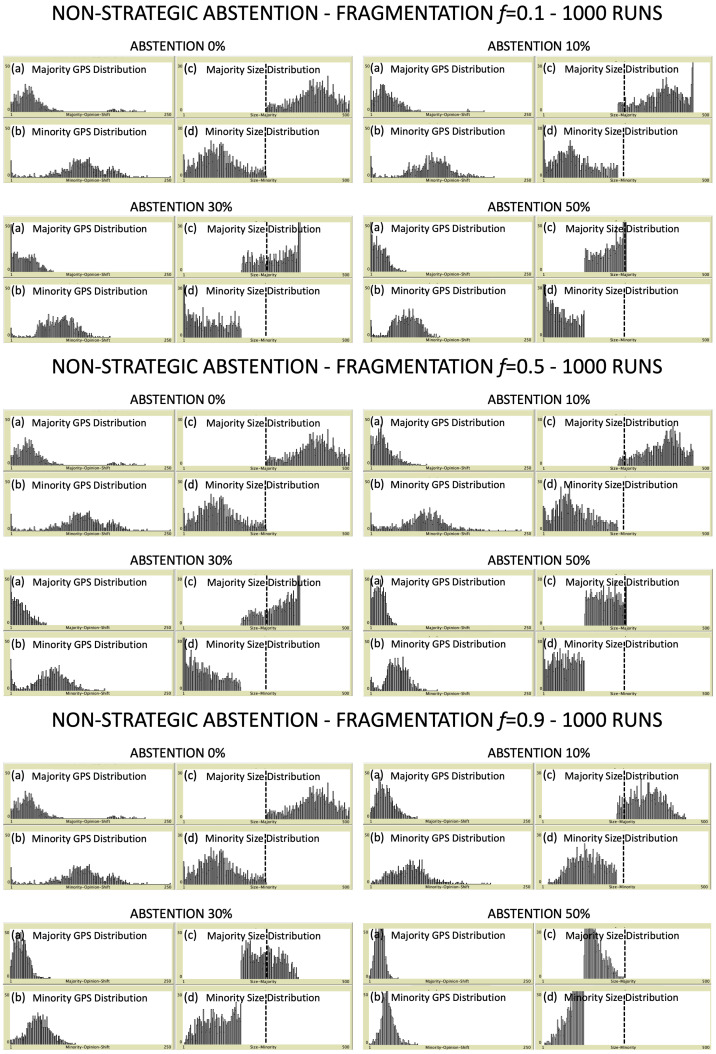
Non-strategic abstention—In correspondence of each fragmentation level and each degree of abstention, the following four panels are reported: (**a**) majority gps distribution; (**b**) minority gps distribution; (**c**) majority size distribution; (**d**) minority size distribution. Output data are collected over 1000 simulation runs. Scale of *y*-axis goes from 0 to 50 for GPS distributions and from 0 to 30 for Size ones. The absolute majority threshold at N/2=250 voters is reported in panels (**c**,**d**) as a vertical dashed line. See text for more details.

In Figure 2 and Figure 3, we plot the gps distributions for both majority (panel a) and minority (panel b), and the distributions of their relative sizes $N_{M}$ and $N_{m}$ (panels c and d, respectively). In the latter panels, a vertical dashed line placed at N/2 voters helps to recognize the threshold for having absolute majority. In the case of strategic abstention (Figure 2), changes in shape for both majority and minority gps distributions are difficult to be appreciated at a first sight, irrespectively of both fragmentation levels and abstention degrees. On the other hand, as expected, size distributions of majority and minority are always non overlapping, and progressively shift on the left when the percentage $p_{A}$ increases, until both of them result to be included within the left half of the plot. Such a result shows that the higher the abstention degree is, the lower the fraction of simulation runs which ends with absolute majority. No evident differences emerge for different fragmentation levels. Size distributions show a similar behavior in the case of non-strategic abstention (Figure 3). However, for increasing degrees of abstention, an evident change in the shape of gps distributions can be appreciated for both majority and minority. In fact, the distributions tend to be compressed and, at the same time, to move on the left, towards lower values of the global preference shift. This effect is even more pronounced by increasing the fragmentation level. As last comment about Figure 2 and Figure 3, it is worth to notice that, in general, the considerable width of the gps distributions for both majority and minority makes them overlapping for any combination of control parameters. Anyway, as we will show shortly, the average gps values of the two coalitions always remain well separated: thus, being the shape of the distributions quite symmetric, we will be able to extract useful information from averages without considering standard deviations.

**Figure 4:**
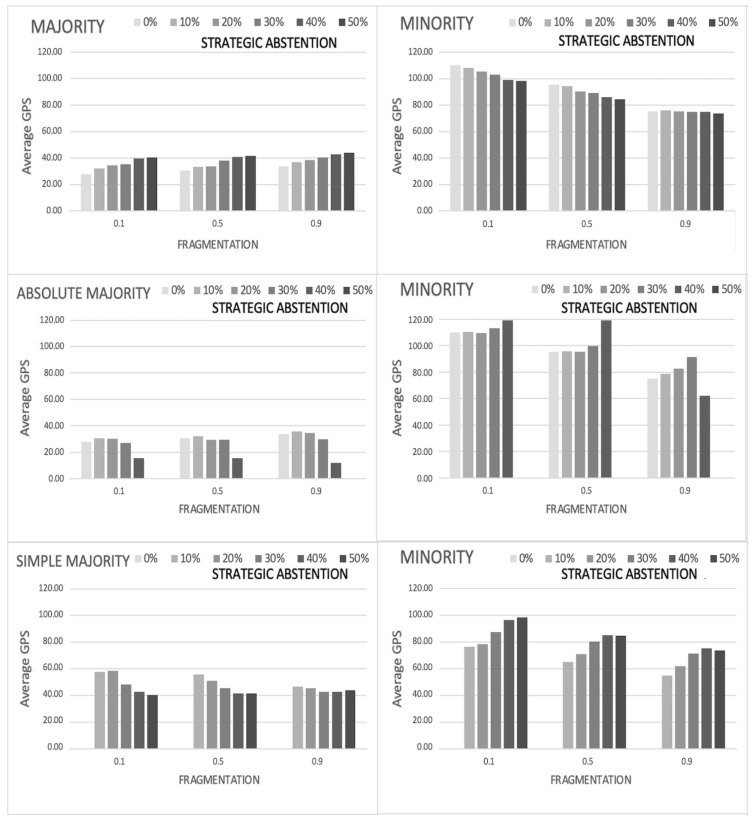
Strategic abstention—Average gps as a function of political fragmentation and degree of abstention, for majority (**top left** panel) and minority (**top right** panel). The same quantities disaggregated for cases when absolute or simple majorities are reached, are also reported in the middle and bottom panels, respectively. With a few exceptions, the effects of abstention on minority seems always positive in terms of preference representation (average gps reduction), for any level of fragmentation. Disaggregated data show an apparent countertrend (an increasing minority gps) which can be explained through statistical considerations about the relative proportion of simulation runs giving rise to absolute and simple majorities. See text for more details.

**Figure 5:**
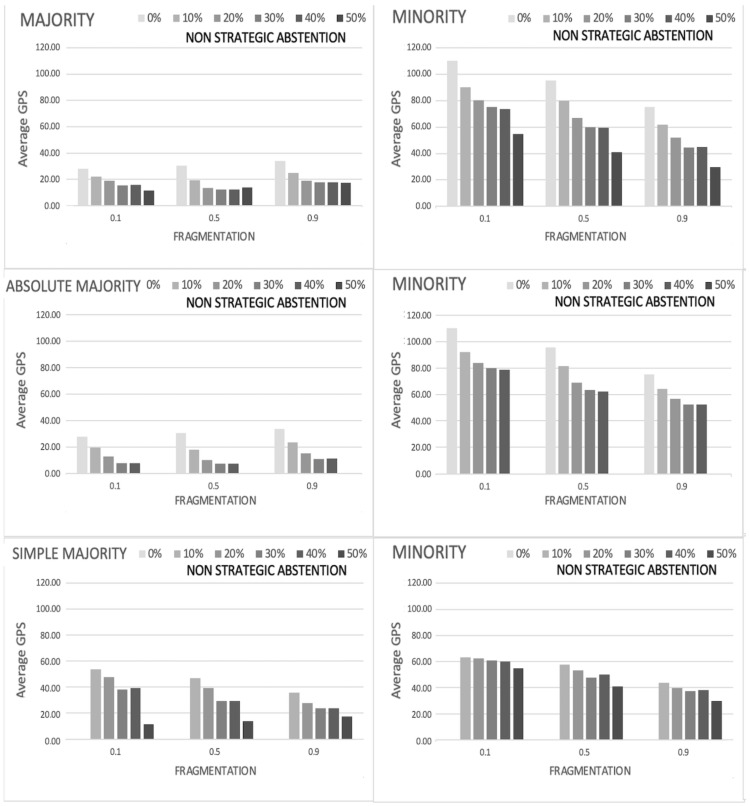
Non-strategic abstention—Average gps as a function of political fragmentation and degree of abstention, for majority (**top left** panel) and minority (**top right** panel). The same quantities disaggregated for cases when absolute or simple majorities are reached, are also reported in the middle and bottom panels, respectively. The effects of abstention on minority seems always positive in terms of preference representation (gps reduction), for any level of fragmentation. No countertrends in disaggregated data is observed for this type of abstention. See text for more details.

In order to better interpret the previous results about the global preference shift, we compare the average gps for the majority and minority coalitions as function of the political fragmentation and of the degree of abstention for both strategic and non-strategic abstention, as illustrated, respectively, in the top panels of Figure 4 and Figure 5. Three main results emerge from this comparison.

Firstly, the effects of abstention on minority are always positive in terms of preference representation (i.e., it always produces a decrease of the average gps), with the sole exception of strategic abstention for an high level of political fragmentation (f=0.90), whose values are almost constant irrespectively of the abstention levels. In other words, voter abstention seems to contrast tyranny of majority and better guarantee representation of minorities. Looking more closely to the results, the positive effect of abstention is quite higher in the case of non-strategic abstention than in strategic one. When voters peripherally located in the preference space decide to remain out of the political debate, as it is the case for non-strategic abstention, the final decision is not affected by extremist positions and benefits both minority and majority, who are expected to be more close in terms of preference setting. Better, the higher the abstention rate, the closer the minority is to the preference profile of majority. A different scenario arises in the case of strategic abstention. When abstention occurs for voter who are uniformly distributed, the final decision appears to be more contestable so that the abstention does not change so much the composition of minority to make it closer to the final decision.

Secondly, such a process is well designed by the evolution of the average global preference shift of the majority, which is the second evidence of the simulation. Majority responds differently to strategic and non-strategic abstention. In the first case, although slowly, abstention reduces the preference representation (increases the average gps) as a reaction to the risk of being contestable, by adopting a final solution someway closer to the minority. By contrast, in the case of non-strategic abstention, for any level of fragmentation, the average gps slowly decreases according to the degree of abstention. Evidently, the absence of extremist positions in the active voting favors a final solution close to the majority, whose average global preference shift is noticeably lower than in the case of strategic abstention.

Finally, political fragmentation results to be a key characteristic to better capture the majority response to abstention. Specifically, a pronounced political fragmentation coupled with abstention generally improves the effective qualification of democracies, thus reducing the risk of tyranny of the majority.

Some further information can be extracted by disaggregating the previous results for absolute and simple majorities, as shown in the middle and bottom panels of Figure 4 and Figure 5. Although for non-strategic abstention (Figure 5) the behavior of the average gps just reproduces what already observed in the aggregated situation for absolute and simple majorities, however, when strategic abstention is considered (Figure 4), a counter-intuitive effect does appear. Regardless the fragmentation level, the average gps for absolute and simple majorities show an inverted trend with respect to the aggregated one: for majority it decreases with the degree of abstention, and the opposite occurs for minorities. This unexpected behavior can be explained by noticing that, as already shown in Figure 2, the relative proportion of absolute and simple majorities over the 1000 simulation runs does not remain constant when the percentage $p_{A}$ increases (for details see also Table 1, introduced below). For small values of abstention most of the runs give rise to absolute majorities, which tend to penalize minority representativeness (average gps is always above 100). For high values of abstention almost all the runs give rise to simple majorities, which tend to benefit minority representativeness (average gps is always below 100). The aggregated behavior, which show the already discussed decreasing trend of minority gps for increasing values of abstention, does emerge from a balance between these two opposite behaviors, favored by the uniform distribution of strategic abstained voters over the preference space. On the contrary, the peripheral distribution of non-strategic abstainers evidently represents an obstacle for such a process, since it reduces the preference space allowed for the consensus dynamics thus hindering the previous phenomenon.

The previous conclusions are further corroborated by Table 1, which shows majority and minority quantitative details for the two types of abstention. All the reported values have been averaged over the 1000 simulation runs. First, the behavior of the size $N_{M}$ of majority as function of the degree of both the kind of abstention (first group of three columns) confirms the transition from absolute majority ($N_{M}$ > 250) to simple majority ($N_{M}$ < 250) for high values of $p_{A}$, with a slight dependence on the fragmentation level *f*. Moreover, it can be seen that the size reduction of the two coalitions as a function of the abstention rate is not symmetric. Indeed, the ratio between the sizes of majority and minority (second group of three columns) decreases for strategic abstention, but increases for the non-strategic one (except for 50%), regardless of fragmentation. At the same time, the number of clusters composing the minority (last group of three columns) tends to increase for the strategic abstention, specially in the case of a strong political fragmentation, and is almost constant for the non-strategic one. Hence, the majority appears to be more ‘contestable’ in presence of strategic abstention, which may foster the formation of new alternative majorities. When less informed indifferent voters strategically prefer abstention rather than voting, it is likely that the risk for majority to be contestable becomes higher, so that the final decision goes progressively closer to minorities.

## 4. Conclusive Remarks

In this paper, we presented a modified version of the Hegselmann and Krause model in two dimensions, in which the debate and all subsequent phases of political agreement have been implemented in order to study consequences of abstention on democratic voting systems. Additionally, we also introduced political fragmentation to better capture the framework in which voting process occurs. The main focus is about the role of both strategic and non-strategic abstention on minority representativeness. Specifically, we believe that, within the limits of the explored parameter space, our results could help to shed light on the existing debate on the potential risk of tyranny of majority. In fact, regardless of the typology of abstention, we found that minorities always benefit from voters’ abstention, while majorities only gain from the non-strategic abstention, independently of the political system fragmentation. These results encourage us to believe that the proposed model, specifically designed to explore the dynamical process driving towards a final decision in a two coalitions political system, could surely be considered for further investigations in this context, possibly extending the parameter space.

## Figures and Tables

**Table 1:** The four main columns, further classified according to values of fragmentation, report the effects of both types of abstention (for increasing values of $p_{A}$) on, respectively, the size $N_{M}$ of reached majority, the ratio between the sizes of majority and minority ($N_{M}/N_{m}$), the size $N_{m}$ of the minority, and the number of clusters forming the minority. Notice that, regardless of fragmentation, $N_{M}/N_{m}$ decreases for strategic abstention, but increases for the non-strategic one (except for 50%). Values are always averaged over 1000 simulation runs. See text for more details.

	av size maj			maj/min ratio			av size min			av min clusters		
strategic abstention	f value			f value			f value			f value		
	0.1	0.5	0.9	0.1	0.5	0.9	0.1	0.5	0.9	0.1	0.5	0.9
0%	393	375	340	3.67	3.00	2.13	107	125	160	2	4	11
10%	342	331	301	3.17	2.78	2.02	108	119	149	2	5	12
20%	300	285	262	3.00	2.48	1.90	100	115	138	2	5	12
30%	259	245	224	2.85	2.33	1.78	91	105	126	2	5	13
40%	215	204	187	2.53	2.13	1.65	85	96	113	3	6	14
50%	177	166	153	2.42	1.98	1.58	73	84	97	3	6	14
	**av size maj**			**maj/min ratio**			**av size min**			**av min clusters**		
**non** **strategic** **abstention**	f **value**			f **value**			f **value**			f **value**		
	**0.1**	**0.5**	**0.9**	**0.1**	**0.5**	**0.9**	**0.1**	**0.5**	**0.9**	**0.1**	**0.5**	**0.9**
0%	393	375	340	3.67	3.00	2.13	107	125	160	2	4	11
10%	362	357	315	4.11	3.84	2.33	88	93	135	2	4	11
20%	329	333	287	4.63	4.97	2.54	71	67	113	2	3	9
30%	294	293	247	5.25	5.14	2.40	56	57	103	2	3	8
40%	292	292	248	5.03	5.03	2.43	58	58	102	2	3	8
50%	208	187	157	4.95	2.97	1.69	42	63	93	3	4	10
